# Making a Difference: Planning for Engaged Participation in Environmental Research

**DOI:** 10.1007/s00267-021-01585-5

**Published:** 2022-01-09

**Authors:** Daniel B. Ferguson, Alison M. Meadow, Henry P. Huntington

**Affiliations:** 1 Department of Environmental Science, University of Arizona, Tucson, AZ USA; 2 Arizona Institutes for Resilience, University of Arizona, Tucson, AZ USA; 3 Huntington Consulting, Eagle River, AK USA

**Keywords:** Transdisciplinary approaches, Engaged participation, Research outcomes, Maturity of relationships, Context knowledge, Intensity of effort

## Abstract

Despite the rapid and accelerating rate of global environmental changes, too often research that has the potential to inform more sustainable futures remains disconnected from the context in which it could be used. Though transdisciplinary approaches (TDA) are known to overcome this disconnect, institutional barriers frequently prevent their deployment. Here we use insights from a qualitative comparative analysis of five case studies to develop a process for helping researchers and funders conceptualize and implement socially engaged research within existing institutional structures. The process we propose is meant to help researchers achieve societal as well as scientific outcomes relatively early in a project, as an end in itself or en route to greater engagement later. If projects that have a strong foundation of dialog and shared power wish to use TDA within current institutional and academic structures, we suggest that they focus on three process-based factors to increase their chances for success: (1) the maturity of relationships within a collaboration, (2) the level of context knowledge present within the collaborative team, and (3) the intensity of the engagement efforts within the project.

## Introduction

Climate change, landscape changes from human activities, widespread species extinctions, and increasing levels of environmental contamination are among the litany of challenges threatening the social and ecological systems that humans rely upon (IPBES 2019; IPCC 2018). The real-world impacts of these threats put environmental researchers in an interesting position: our research is theoretically highly relevant and potentially useful for addressing these challenges, yet much of the work we do is divorced from the societal contexts in which it may be useful. This well-understood challenge has led to a significant amount of scholarship and practical work to reduce the disconnect. A central tenet of this socially relevant, problem-oriented environmental research movement is that engagement between researchers and those who have a stake in their work is critical if the research is to have impact on real-world challenges. Direct and iterative engagement is commonly described as the primary means for producing knowledge that is usable or actionable for addressing complex environmental issues (Dilling and Lemos 2011; Fazey et al. 2014; Pohl 2008).

As described in the extensive interdisciplinary literature on the topic, societal engagement is focused on building collaborative relationships between scientists and their partners in the broader society to co-develop knowledge meant to address a shared problem or issue (Lemos and Morehouse 2005; Reed et al. 2014). A variety of collaborative approaches and terms are utilized in contemporary environmental research including: co-production of knowledge (Lemos and Morehouse 2005; Meadow et al. 2015); development of actionable science (Bamzai-Dodson et al. 2021; Beier et al. 2017); Mode 2 science (Gibbons et al. 1994; Nowotny et al. 2003); post-normal science (Funtowicz and Ravetz 1993); and many others^1^. In moving beyond disciplinary research, scholars have distinguished between various approaches meant to bring different ways of knowing together. Tress et al. (2005), for example, delineate three broad categories of integrated research: *multidisciplinary* (different disciplinary actors working on a shared goal with differing disciplinary objectives), *interdisciplinary* (different disciplines integrate their knowledge to generate new knowledge and theory focused on a common research goal), and *transdisciplinary* (different disciplines working with non-academic partners to create new knowledge and theory aimed at a shared question). In a recent review Knapp et al. (2019) situated what they call “transdisciplinary approaches” or TDA in pre-existing socially-engaged research approaches. They define transdisciplinarity as a research mode that “connects diverse knowledge holders with one another and the realm of practice, shares power within the process, and arrives at different outcomes including action and problem management” (Knapp et al. 2019, 2). Despite their promise, TDA have proven to be extremely challenging to implement (Brandt et al. 2013; Cvitanovic et al. 2015; Lang et al. 2012).

Large scale efforts to move institutions and researchers toward TDA for global change knowledge development like Future Earth (Leemans 2016; van der Hel 2016) represent promising opportunities for bringing about the kinds of social transformations frequently called for by scholars who study transdisciplinarity. However, the pace of environmental change and impacts from those changes is outpacing the rate of institutional change required to bring widespread use of TDA to scale globally. We strive to help bring about faster practical change by examining the early stages of socially engaged research. Our intention is to empower environmental scientists who wish to have as much societal impact as they can, but may be daunted by the length and difficulty of TDA as too often experienced by both researchers and societal partners (Cvitanovic et al. 2019). In addition to helping researchers achieve practical impacts in their work and begin the journey toward using TDA, we hope that our ideas will also help funders and research institutions recognize what can be achieved in the early stages of TDA and value the impacts that can be made early on. To that end, we have compiled a set of concepts from the literature and our own work that inform a process for conceptualizing and carrying out transdisciplinary environmental research within existing institutional structures. Consistent with the literature on TDA, our process rests on a foundation of shared power and dialog to develop a set of shared aims. Building on those foundational concepts, we propose that TDA can be productively utilized despite current barriers if researchers give more deliberate attention to three process factors we found important in our review: (1) the contextual knowledge of the collaborative team, (2) the strength of interpersonal relationships within the team, and (3) the intensity of engagement necessary for genuine collaboration to occur.

Because there are numerous transdisciplinary environmental frameworks in the literature (e.g., Daniels et al. 2020; Hoffmann et al. 2019; Jahn et al. 2012; Pohl et al. 2017), our goal is not to offer another framework, but rather to build on that robust theoretical and empirical foundation to present a practical process that offers researchers and funders a realistic way to engage in socially-impactful environmental research. Our motivation is threefold. First, we aim to make salient insights from the specialized TDA literature accessible to researchers and funders with a broad range of expertise and interest. Next, by doing so we hope to help more environmental researchers and funders be prepared to utilize and support TDA so that critical environmental problems can be addressed more quickly. Finally, by engaging a broader research community in this kind of work, we aspire to contribute to the ongoing movement to get TDA more widely accepted and supported by research institutions and funders.

### Impediments to Transdisciplinary Knowledge Development

A range of barriers limits the utility of TDA for combating the relatively rapid environmental changes communities around the world are confronting. A critical root of these barriers is institutional inertia within academia and many public funding agencies that have persistently limited more widespread adoption of interdisciplinary research (Arnott et al. 2020; Bromham et al. 2016), a necessary precondition for institutional support of transdisciplinarity. Specifically, we note four barriers that are currently preventing more widespread adoption of TDA.

First, universities (and other research-focused organizations) tend to prioritize the production of novel, generalizable knowledge (i.e., basic science published in peer-reviewed literature) over practical, place-based, or “usable” knowledge and associated products and outcomes (Cvitanovic et al. 2015; Cvitanovic et al. 2019; Foster 2010; Gaziulusoy et al. 2016; Kopp 2021). Second, this institutional legacy limits opportunities for researchers to be trained in TDA methods and approaches (Rozance et al. 2020). Third, the academic evaluation system is still largely focused on publication in peer-reviewed journals, a metric that is easier to quantify than societal impacts (Penfield et al. 2014; Alvesson et al. 2017; Spaapen and van Drooge 2011; Meadow and Owen 2021) and that does not place much value on other outputs such as reports, workshops, or services that may be more relevant to societal partners (Kopp 2021). Finally, the prioritization of basic research is often reflected in funding mechanisms that do not adequately support activities such as travel and additional time for meetings and engagement, including support for societal partners’ participation, that are critical to the success of transdisciplinary research efforts (Arnott et al. 2020; Gaziulusoy et al. 2016; Rowe and Lee 2012; Shanley and López 2009).

These (and numerous other) barriers to widespread adoption of TDA are well known, yet remain embedded in the culture of academic research institutions (Houser et al. 2021). We recognize, therefore, that in addition to developing an environmental research workforce that is capable of and comfortable with using TDA, we must simultaneously and consciously utilize the success of that workforce to shift obstructive aspects of the institutional landscape away from academic insularity and toward more socially-engaged environmental research. What we describe below, therefore, is a distillation of concepts central to success in TDA that can be adopted by nearly any researcher who is committed to seeking greater social impact from their work, even if they are still working within the confines of existing institutional structures. The path we outline is neither easy nor an excuse to avoid addressing institutional barriers, but we hope it is an accessible entry point into TDA for those interested in pursuing, funding, and otherwise supporting this type of work.

### Fundamental Principles for Engaged Research

We start with two pillars of TDA that involve genuine engagement between researchers and societal partners: open dialog and shared power.

Although in contemporary environmental research there are now a plethora of more integrative approaches to generating new knowledge, many institutional structures are rooted in less expansive ways of thinking. For example, the concept of a linear model of science (i.e., basic science creates a wellspring of knowledge that can be dipped into for human advancement) presented in Vannevar Bush’s 1945 report *Science, The Endless Frontier* has persisted as a way of conceptualizing how science stands in relation to—and somewhat apart from—the rest of society (Stokes 1997). This model has led to a multitude of challenges for addressing complex problems (Sarewitz 2016) and has helped perpetuate the notion that science can best “speak truth to power” when it is kept above the fray of real-world problems (Jasanoff and Wynne 1998). The linear model, therefore, implicitly endorses a one-way flow of information from science to society, or what Rowe and Frewer (2005) call “public communication.” Rowe and Frewer also describe one-way information flow in the other direction, or what they call “public consultation.” Regardless of direction, one-way information flow prevents opportunities for dialog and therefore has limited utility for finding the common ground necessary for collaborative work to address complex, socially-relevant problems. It is only when information flows in both directions that “public participation” can occur (Rowe and Frewer 2005).

Talwar and colleagues add nuance to this basic premise in their framework that divides what they call “unidirectional social research” from “interactive social research,” where the latter begins when non-academic partners become engaged in designing the research strategy (2011, 381–382). Dilling and Lemos refer to the two-way interaction between researchers and information users as iterativity or “purposeful and strategic interaction between…knowledge producers and users” and argue that it is necessary “to increase knowledge usability” (2011, 681). Kassam (2015) notes that this intentional dialog adds both cultural and cognitive diversity, which he argues is necessary for addressing highly complex, socially relevant problems like those associated with environmental degradation. This line of scholarship responds to traditional notions of scientific engagement that has been focused on *talking to* society and argues that that for complex decision contexts to truly benefit from research, scientists must consciously *collaborate with* partners in the broader society, a concept Steger et al. call “science with society” (2021). Research that utilizes TDA requires dialog in part because successful transdisciplinary projects require the establishment and continued revisiting of the shared goals of the participants (Huntington et al. 2011; Owen et al. 2019; Steger et al. 2021). Without productive two-way communication the aims of the various actors involved may diverge significantly and the project may reflect only those who are most vocal or who hold the most power. Importantly, for researchers working within existing institutional structures, traditional outputs (e.g., peer-reviewed publications) may be necessary so it is critical that researchers—as well as nonacademic partners—acknowledge their specific needs and objectives as shared goals are being established.

We also draw from scholarship that argues that engaged research requires that relevant stakeholders be included as partners in these collaborations and share power and decision making with researchers. Citizen engagement in decision making has been categorized by Arnstein (1969) as a ladder, with only the top three of eight rungs (partnership, delegated power, and citizen control) reaching citizen power. Biggs (1989) identified four modes of research based on the amount of local knowledge and participation involved in each mode. The modes ranged from contractual, which has limited engagement, through collegial, in which local knowledge and knowledge holders are full and equal participants, with active attention to the strengthening of local knowledges. David-Chavez and Gavin (2018) build on Biggs to add a highly engaged mode of interaction they call *Indigenous*, which centers research in Indigenous community values and research methods.

Recently within academia many scholars from historically excluded communities have made critical contributions to the participatory research literature, in part by challenging the status quo power imbalances between western-trained environmental researchers and marginalized communities (see, e.g., David-Chavez and Gavin 2018; Jäger et al. 2019; McGregor 2014; Tuhiwai Smith 2012; Whyte 2017). Much of this scholarship is specific to Indigenous communities, but the broader points that emerge—centering of local needs and values, ethical participatory research practice, respectful collaboration, humility—are all highly relevant to any environmental research using TDA (Wilmer et al. 2021). This point is reinforced by Steger et al. (2021) who surveyed 168 people involved in environmental transdisciplinary work worldwide and found that unequal power dynamics between researchers and community partners was the top barrier to successful transdisciplinary research reported by non-researchers (2021).

Finally, keying in on the power imbalances that can subvert the intention of participatory research practice, the primary distinction public health researchers Goodman and Thompson (2017) make between approaches that are effectively one-way (from science to society) and those that are bi-directional is the extent to which non-academic partners have a measure of power and control over the direction of the work. In their framing, the three primary criteria for a project to fall into their “engaged participation” category are: both researchers and community members are actively involved in the design and implementation of the project as well as interpreting the findings, everyone involved benefits somehow from collaborating, and responsibility for decision-making and resource allocation is collaborative with a focus on equitable distribution of power and valuing of community input (Goodman and Thompson 2017, 487).

We focus on Goodman and Thompson’s “engaged participation” for the remainder of this paper because it allows us to consider collaborations between researchers and non-academic partners that entail shared power and responsibility, but still may not achieve the level of integrated and co-produced knowledge commonly seen as the most challenging aspect of transdisciplinarity (Jahn et al. 2012). As with the framework presented by Bamzai-Dodson et al. (2021), we seek to offer practical insights that allow researchers and their partners flexibility to design impactful research that can achieve the specific goals relevant to the collaboration.

### More Nuanced Measures of Impact in Transdisciplinary Approaches

Demonstrating that a project has had a beneficial societal impact is one of the challenges of engaged participation research. Older models of information use often focused on or privileged instrumental uses (i.e. information is used to directly inform a new decision or action) based on assumptions that input of information into an organization would lead to the output of use of that information (for a review of information use theory, see VanderMolen et al. 2020). Eventually greater nuance was added to information use models, such as when Pelz (1978) proposed three categories of use: instrumental; conceptual, where an agency or individual is better informed of an issue; and symbolic (also termed legitimative or justification), where information is used to substantiate a decision already made. Notably, empirical studies have found that conceptual uses are far more common than instrumental (Amara et al. 2004; Nutley et al. 2007) and can be critical stepping stones required to reach more tangible impacts (Nutley et al. 2003; Oh 1996). Failure to recognize conceptual impacts can leave gaps in our knowledge about the efficacy of our research and engagement practices.

As the field of research evaluation has wrestled with the question of how academic research has impacts in the world, there has been greater recognition of the need to understand the process by which knowledge is produced—alongside evaluation of its use by and impact on societal actors (Spaapen and van Drooge 2011; Muhonen et al. 2020). For example, Meagher and Martin (2017) and Edwards and Meagher (2020) added new categories to the existing use and impact models particularly focused on conceptual impacts such as: capacity building impacts (training and/or developing collaborative abilities, roughly equivalent to the National Science Foundation’s broader impacts definition); enduring connectivity (establishment of long-lived external relationships); and attitude or cultural change (increased willingness to engage in knowledge exchange activities, on the part of individuals, institutions, or organizations). Edwards and Meagher (2020) describe capacity-building, attitude change, and enduring connectivity as specific types of conceptual impacts.

Beyond questions of definition of what is, or is not, an impact, we face challenges in documenting and articulating those research impacts, particularly the less tangible conceptual impacts. Despite the challenges, significant work is occurring within the field of research evaluation, particularly as more government funding bodies seek to understand the societal return on their investments (Bayley and Phipps 2019; Meadow and Owen 2021). One challenge is the significant time lag between the emergence of research results and when practitioners adopt new practices (Bell et al., 2011; Penfield et al., 2014; Spaapen & van Drooge, 2011). In medical research, this lag can be up to 17 years (Bolas and Boran in Moore et al. 2016). The lag can be attributed to whether an organization is prepared and able to integrate new knowledge, the level of uncertainty surrounding the new knowledge, and the pertinence of the new knowledge to the specific needs of the practitioners (Ford et al. 2013; Green et al. 2009; Oh and Rich 1996; Oliver et al. 2014). This time lag makes tracing conceptual uses all the more important because it allows researchers to learn what resonates with their societal partners relatively early in the research process. Another prominent challenge is attribution of any one impact to a specific piece of research or research process (Bell et al., 2011; Boaz et al., 2009; Penfield et al., 2014; Spaapen & van Drooge, 2011; Wiek et al. 2014). Contribution analysis (Morton 2015) can shed light on the role played by a particular research effort, while also elucidating the other factors that supported or hindered uptake of new knowledge.

Both of these challenges can be reduced by the inclusion of ongoing and embedded evaluation in research projects, which allows researchers to collect feedback from societal partners as a process progresses, reducing memory lapses, recall bias, or the impact of participant turnover (Wall et al. 2017).

## Methods

### Development of Factors to Consider in Designing Engaged Participation Projects

To arrive at the process we offer below, we chose to closely examine five case studies, four of which we were directly involved with and one (the Colorado River study) that was carried out by a group of close collaborators of two of the authors (Ferguson and Meadow). We considered these cases in the context of the existing literature and selected them based on: our familiarity with the engagement processes used and the outputs and outcomes of each; the explicit focus within each on socially-relevant goals; the time that had elapsed since the projects had formally ended so that we had some distance to consider longer-term impacts. Through conversations that reflexively (Creswell and Creswell 2020) considered our prior knowledge of and investment in these projects, we designed a qualitative assessment template^2^ in which we entered details about each case in four categories: project background (e.g., who was involved, who initiated the project, had the collaborators worked together previously, when did it take place), what level of engagement took place (e.g., types of and frequency of communication and meetings), what level of “context knowledge^3^” existed (e.g., prior experience the collaborators had working in their partner’s—practitioner/academic—domains and the nature of that experience), and outputs and outcomes from the projects. Gathering outputs (e.g., reports, peer-reviewed publications, datasets) and tangible outcomes (e.g., use of project outputs in decisions, collaborations beyond the project, new research questions and funding) was straightforward. Because this was not a formal evaluation project, detailed data gathering about less tangible outcomes was beyond the scope of our work here. Instead, we chose to include in each case template researcher reflections on how the project impacted their thinking as well as how they perceived the work impacted their partners. For the case that none of the authors worked on, one of us (Ferguson) worked with the project’s lead investigator to fill out the case template. For the other four cases, the involved author filled out the case templates.

Once the templates were completed and assembled into a common spreadsheet, the authors iteratively worked through the information in a process of abductive reasoning—or “working back and forth from an observed consequence to a probable antecedent or cause” (Teddlie and Tashakkori 2009, 89)—to better understand how this set of socially engaged research projects functioned. That process made clear that a common feature was that each demonstrated conceptual and/or instrumental impacts within the first couple of years of the work, which led us to look for other common features in the research processes that might help to explain the generation of impacts in a relatively short time. Our observations and reasoning suggest three factors that influence the degree to which projects achieve positive socially-relevant impacts: 1) the relative maturity of the relationships within the partnership, 2) the intensity of the engagement effort, and 3) the extent of context knowledge within the team^4^. The first two are drawn from literature, as we describe below. The third arose from considering our own work and the specific projects we describe below. What we present here, therefore, is what Agar (2013) calls a “rational reconstruction” that uses our experiences as researchers to point to possible explanations for the patterns we observed.

We primarily looked at the early part of these collaborative projects—approximately the first two years—for three reasons. First, many research projects are funded for relatively short periods, only a few years. We wanted to examine ways in which even these short-term projects could be structured to cope with some of the institutional barriers noted above and yield socially relevant outcomes on a short timeframe. Second, short-term successes may be indicators that projects will develop into longer-term transdisciplinary processes through time. Finally, our two-year focus was supported by Kothari et al.’s (2011) framing of early and mature partnerships in which they noted that the outcomes of partnerships tend to become more tangible when partners have been working together for approximately two years. Our primary interest here is in the payoffs that occur early on, rewarding even initial efforts that may go no further or that may stimulate those involved to invest in further efforts.

We recognize that there are many factors both within and outside of a project and/or team that could influence the outcomes and impacts of any given project. We also recognize that formal evaluation of climate research projects that utilize TDA is a robust and growing field (Ferguson et al. 2016a; Meadow et al. 2013; Meadow et al. 2016; Meadow and Owen 2021; Owen et al. 2019; Wall et al. 2017). Our goal here was not to formally evaluate these projects to document specific impacts. Instead, we consider this an effort to lay conceptual groundwork for future research on these (and other) factors and to encourage researchers to consider the role these factors play in their research as we collectively work to improve our practice and speed the pace of actionable solutions to environmental problems. Below we describe the three factors we identified in greater detail and how we arrived at them. Readers interested in considering these factors in their own work may use the descriptions below as well as the questions included in the supplemental information.

#### Maturity of Relationships

As noted above, Kothari et al. found that “partnerships within their first 2 years of coming together exhibited different characteristics than those partnerships of an earlier vintage” (2011, 207). For example, they found that “meeting information needs, the level of rapport and the commitment to the partnership were more pronounced in mature partnerships” (Kothari et al. 2011, 209). Similarly, Austin describes four phases that individuals involved in participatory anthropology partnerships pass through from an early phase of getting acquainted and building relationships through close cooperation when research and innovation occurs to consolidation and “productive coexistence” when relationships truly mature and finally to the termination of the partnership (2004, 422) (Austin 2004). One reason the maturity of relationships is so significant is the necessity of building trust among all involved, which takes time and sincere and respectful interactions (Grant et al. 2008).

For our purposes, we conceptualize the maturity of partnerships as reflecting the relationship between the people involved, *not the length of the project at hand*. Therefore, we define an early partnership as one in which there are no pre-existing relationships between researchers and stakeholders. Mature partnerships, then, are those built around pre-existing relationships. For the purposes of our cases, we simply delineated between those who had prior collaborative relations (we considered those “mature”) and those that did not. A project may be new, but the relationships and trust between researchers and partners have been well-established through previous projects or interactions. As discussed above, the work of Kothari et al. (2011) suggest that relatively young partnerships would yield fewer examples of instrumental uses of the research by societal partners.

#### Intensity of Engagement

In their typology of user engagement strategies in sustainability research, Talwar et al. (2011) emphasize that when projects rise to what they call “interactive social research” there is a “leap in intensity [that] fundamentally shifts the engagement into two-way interaction: away from a situation in which the users serve as audience or subjects of the research” (382). The increasing effort required to maintain collaborative relationships, attend to power imbalances, and ensure clear communication among partners is commonly seen as one of the basic challenges of carrying out engaged research (e.g., Gaziulusoy et al. 2016). In our initial case selection, we specifically sought projects that we felt met the standard of interactive social research described by Talwar et al. (2011).

Because simple counting of occurrence is an imprecise measure of intensity of engagement, to assess our selected cases we considered both the *frequency* and *quality* of communications (Blackstock et al. 2007; Wiek et al. 2014) and joint convenings, for example meetings and workshops (Blackstock et al. 2007; Walter et al. 2007; Wiek et al. 2014). Frequency can include both the number of meetings and also the length. Quality can include factors such as the degree to which meeting agendas were jointly developed, the number of people involved from each participating group, how ideas at the meeting were (or were not) put into practice, and similar ideas. We also looked for the presence of jointly produced outputs, such as fact sheets, reports, policy briefs, peer-reviewed publications, data sets, and websites (Reed et al. 2014; Wall et al. 2017). Taken together these aspects of a project provided a simple way to consider how much effort was spent on engaged participation relative to more traditional research activities and products, such as writing scientific papers or giving presentations at academic conferences, where societal engagement is less likely to be a key feature.

#### Context Knowledge

As we worked back and forth between our cases and the literature we recognized that a factor that we observed and felt was important, but which we had not seen well articulated, is the extent to which participants in a collaboration had a broad contextual knowledge beyond the disciplinary expertise they brought to the project^5^. For our purposes, we define context knowledge as: *insightful understanding of the particular circumstances in which new information may be generated and applied*. We assume a researcher will always bring *content* knowledge—or expert insights about their own research domain— into a collaboration, but not all researchers will have *context* knowledge that allows them to understand how their research may be relevant or useful—and to whom—in a policy, management, or other decision-making situation. The same can be said for non-academic collaborators: we assume they will bring *content* knowledge about management, policy, or other domains to a partnership, but may lack *context* knowledge about the academic research process or about the specific research domain in which the collaborative problem resides.

We recognize that context knowledge may not always be a positive factor within a team (e.g., when context knowledge brings along implicit or explicit bias), but it is outside the scope of this manuscript to attempt to categorize the normative role context knowledge may play in our projects. Instead we chose to qualitatively examine each project for three characteristics that showed the relative levels of context knowledge within the teams: the amount of time researchers/practitioners have engaged in affairs in the region where the project takes place (as distinct from their relationships with individuals as described in “Maturity of relationships” above), the extent to which the involved researchers/practitioners have carried out prior collaborative projects in a similar domain (e.g., location, topic area, etc.), and prior work/life experience that lends insight into academic vs. practitioner cultures (e.g., a former academic who’s now a practitioner or a practitioner who has become an academic).

## Case Studies: Illustrations of Engaged Participation in Practice

To illustrate these factors, we present five brief case summaries of engaged research. The projects we selected have several features in common, including:

- a deliberate strategy to engage with practitioners or decision makers in genuine dialog and empower those nonacademic partners so that each project met the minimum threshold of “engaged participation” from Goodman and Thompson (2017) described above;
- some member(s) of the research team had prior experience with engaged research;
- the project is either completed or has become an ongoing collaboration that grew out of the early engagement phase; and
- demonstrable outcomes that emerged from the first two years of the project (thus we did not include projects that failed to achieve this goal).

The cases—summarized in Table 1—were chosen to include a diversity of collaborators (Indigenous communities, western and Indigenous natural resource managers, a broad range of environmental researchers) and topics (water resources, fisheries, climate and weather, environmental program priority setting). Two were intended chiefly as short-term projects, whereas the other three had at least an aspiration to be long-term efforts (though two of these ended within a few years of starting). All sought to achieve socially relevant outcomes of one kind or another.

**Table 1:** Summary of case studies

Project	Collaborators	Question(s)?	Project start	Project end
Planning for Drought in the Warming and Drying Southwest	Social and physical scientists; Tribal government resource managers	How can drought be characterized with minimal instrumentation in such a way that it can inform government decisions?	2009	2016
Silalirijiit: Studying Weather in Clyde River, Nunavut	Visiting social and physical scientists; one resident social scientist; local community members	How is the weather/wind changing around Clyde River and what does that mean for the locals?	2009	Ongoing
The Northwest Arctic Borough Science Program	Scientists from many disciplines; local residents in NW Alaska	What science would be useful to Borough residents, especially in regard to evaluating development projects?	2014	2016
Examining the Influence of Temperature and Precipitation on Colorado River Water Resources	Physical scientists; federal, state, and local water management practitioners	What is the role of air temperature in CO river streamflow?	2014	2016
Resilience and Adaptation to Change in the Arctic, Alaska, Japan, Norway	Marine scientists from Japan, Norway, Alaska; stakeholders from all three locales	What changes can be expected in Arctic/subarctic marine ecosystems? How will those changes affect fisheries? How can fishers and fisheries managers respond?	2016	2018

The case summary narratives below highlight the three factors we are interested in and the outcomes that emerged from each project within the first couple of years of the collaboration. For each case, we qualitatively scored each factor with simple binaries (high/low; early/mature) based on the information we had gathered about each case (summarized in Table 2). Within each narrative case summary we focus on outcomes (changes in behavior, knowledge, skills, etc.) rather than outputs (meetings, workshops, reports, datasets, manuscripts, operational protocols, etc.). In terms of outcomes, we further differentiate instrumental impacts from conceptual impacts. We considered outcomes like capacity-building, connectivity, and attitude changes (as discussed above) to be examples under the broader heading of conceptual impacts.

**Table 2:** Qualitative assessment of example projects

Project	Collaborative Relationships	Engagement Intensity	Context Knowledge	Impacts
Planning for Drought in the Warming and Drying Southwest	Early	High	Low	Primarily conceptual; eventually instrumental
Silalirijiit: Studying Weather in Clyde River, Nunavut	Mature	High	Low	Conceptual and instrumental
The Northwest Arctic Borough Science Program	Early	High	High	Conceptual and instrumental
Examining the Influence of Temperature and Precipitation on Colorado River Water Resources	Mature	Low	High	Conceptual
Resilience and Adaptation to Change in the Arctic, Alaska, Japan, Norway	Early	Low	High	Conceptual

For each factor, simple binaries were used to characterize the cases. Early partnerships are those without pre-existing relationships between researchers and stakeholders. Mature partnerships are those built around pre-existing relationships. Engagement intensity was qualitatively assessed based on frequency and quality of communications and joint convenings as well as presence of jointly produced outputs. Context knowledge assessment is reflective of all collaborators (nonacademic partners and researchers)

### Planning for Drought in the Warming and Drying Southwest

By 2009 the Hopi Tribe—a sovereign, Indigenous nation whose lands are in northeastern Arizona—experienced years of drought that left lasting impacts across their landscapes. That year several conversations between two University of Arizona researchers (including author Ferguson) and managers from the Hopi Department of Natural Resources (DNR) led to a collaboration to investigate approaches for monitoring drought in their large, rural, semi-arid landscape mostly devoid of weather stations (Ferguson et al. 2016b). With no previous relationships between the research team and Hopi DNR personnel we consider this an early collaboration. Although one of the researchers (Ferguson) had prior work with Indigenous communities in the region, he and the rest of the researchers had low context knowledge since at the outset they had limited insight about Hopi DNR operations, the diversity of management objectives they had to balance, nor the social and cultural drought impacts of concern to DNR staff and tribal leadership. Similarly, the DNR staff had low context knowledge in terms of how academic researchers quantify drought conditions and consider social and biophysical system interactions. Because of these limits, the research team designed a relatively high-intensity engagement strategy aimed at: (1) developing shared understanding of the drought monitoring challenge facing the DNR and (2) building relationships to strengthen the collaboration and ultimately produce meaningful outcomes. Engagement activities early in the collaboration included formal/informal interviews and participant observation during in-person visits to the Hopi DNR offices several times each year and one two-week period when Ferguson worked within the DNR offices; routine phone and email conversations; and a joint trip to Washington, DC to meet with federal agency representatives about the state of drought monitoring on Hopi lands. Although at the outset of the project the Hopi DNR partners sought more instrumental outcomes (better data to directly inform decisions), within the first two years of the collaboration the outcomes for Hopi collaborators were primarily *conceptual*: the initial assessment done through the project revealed that the acute drought monitoring challenges facing the DNR were unlikely to be resolved through tribal investments in instruments. By the fourth year of the collaboration, however, an *instrumental* impact for the DNR did occur when a tribal decision to remove livestock from drought-impacted lands was informed by a collaboratively developed qualitative drought monitoring and reporting process (Ferguson et al. 2016a, 2016b). For the research team, the early engagement activities led to a significant *conceptual* outcome: recognition of the mismatch between scientific characterizations of drought and the needs of natural resource managers and elected officials for managing drought impacts. As a result of these insights, the research team has since pursued more focused studies on what they have come to call the “flavors of drought”—different conditions that lead to different types of drought impacts—in the US Southwest (Crimmins et al. 2017) to try to better match the science with the management context in the region.

### Silalirijiit: Studying Weather in Clyde River, Nunavut

Inuit in Clyde River, Nunavut, has observed many changes in weather patterns over the past few decades. An examination of available meteorological data for the area did not match the Inuit observations well (Gearheard et al. 2010). To explore the reasons for this discrepancy, a multidisciplinary research team (meteorologists and social scientists, including author Huntington) worked with several community members (Inuit hunters) to design and carry out a project to better document Inuit observations, better understand Inuit perceptions of weather, and install three remote weather stations at locations selected by the community to gather data on weather patterns throughout the area. The project built on mature relationships (two of the four academics had experience in the community, and several of the Inuit collaborators had experience working with those two academics). The project had high engagement intensity in the form of community meetings and extended joint travel on the land as a team. However, we considered this project to be an example of relatively low context knowledge because the academics with mature relationships still knew less about Inuit conceptions of weather than they knew about the topics they had previously studied together (e.g., sea ice) and the Inuit likewise had not collaborated with meteorologists before. In other words, context knowledge was relatively high about Inuit and research cultures generally, but relatively low with regard to cultural specifics around weather and meteorology, a shortcoming that the strengths of mature relationships and high engagement helped overcome. In the first two years of the project, the team generated a tangible output—near-real-time weather data from the remote stations via satellite (www.clyderiverweather.org)—that led to what we consider an *instrumental* outcome: frequent access of the remote-station data by Clyde River residents as shown through website analytics. Outcomes for the non-Inuit researchers were primarily *conceptual*: a much-improved understanding of Inuit terminology, perception of weather, and other factors involved in local weather-related decisions that allowed them to ask more sophisticated questions and identify important parameters for modeling that would not otherwise have occurred to them. This deeper understanding allowed the academics to develop the concept of “human-relevant environmental variables” that combine different meteorological, geographical, seasonal, and social factors (Fox et al. 2020), a concept underlying a more recent iteration of the project.

### The Northwest Arctic Borough Science Program

The Northwest Arctic Borough, a county-like government in northwestern Alaska, received funding from Shell Oil to create a science program aimed at addressing the needs of Borough residents (predominantly Iñupiat Eskimo), particularly with regard to impacts and benefits of resource development in the region. In 2013, the Borough created a science steering committee, comprising area residents (hunters, community leaders) and scientists (biologists, public health experts, social scientists including author Huntington) from elsewhere in the state. The area residents and the scientists all had experience with interactions of this kind before and in most cases in the region, so context knowledge was high, even if most of the relationships among committee members were early. Engagement intensity was high, based on frequency of meetings and development of jointly produced outputs. The board met in person several times a year to review the state of environmental knowledge in the region and the main concerns of Borough residents, develop a scientific program, and decide which research projects to fund. The board also convened two synthesis workshops to distill information. For the scientists, the initial outcomes of the engagement were *conceptual*: a greater understanding of the concerns of the borough residents, leading to research projects focused on those concerns. For borough residents *conceptual* outcomes included a comprehensible synthesis of the state of scientific knowledge about the Northwest Borough and its environment and rapid funding of research projects addressing some of their concerns. This project demonstrates how a *conceptual* outcome—the sense of shared purpose and common understanding of how the program could help borough residents—can lead to an *instrumental* outcome: a consensus articulation of the type of research that the program should fund, which led to six priority projects being funded. Unfortunately for the science program, Shell Oil ceased its operations in Alaska in 2015, leading to the end of the program, making it impossible to say how the effort would have evolved from its promising start.

### Examining the Influence of Temperature and Precipitation on Colorado River Water Resources

The Colorado River basin in the western United States supplies water for tens of millions of people, provides irrigation for millions of acres of agriculture, and supports a vast network of ecosystems. Drought and climate change threaten availability of water resources, but until recently little research had been done to specifically examine the relationships between air temperatures and streamflow in the Colorado River. This project sought to better understand these relationships in the Upper Colorado River Basin (McAfee et al. 2017; McCabe et al. 2017; Woodhouse et al. 2016), which is responsible for providing the majority of water to the system, primarily through snow-melt. The study’s central question emerged from interactions between the research team (made up of physical scientists) and water management professionals in the region. The lead researcher (a paleoclimatologist) had approximately 15 years of experience working in the Colorado Basin prior to the project, most of which involved collaborative projects with the water management community. The project was initially funded for two years (2014–2016) and involved the scientists working collaboratively with water resources agencies via a project advisory committee. We consider this project an example of a mature partnership since the project lead and some members of the project advisory committee had worked together for many years. Context knowledge among members of the collaborative team was high because of the lead researcher’s extensive experience working with the water management community and the members of the project advisory committee’s experience funding and otherwise contributing to climate and hydrologic research. We consider the engagement intensity of this project relatively low, though it still met the basic threshold of Goodman and Thompson (2017) engaged participation since there was shared governance and accountability throughout the project’s life and project outputs were designed to satisfy both the management and academic partners’ needs. As a result of engaging with water managers, researchers were introduced to a concept relevant to that community:“runoff efficiency” or the ratio of runoff to precipitation. The analyses that the team conducted eventually used runoff efficiency as a primary metric, increasing the relevancy of their work to water managers. The outcomes of the engaged participation aspect of this project were *conceptual*: both the researchers and the water managers came to a new understanding—both shared and unique to researchers and practitioners—of the relationships between air temperature and streamflow and how those relationships are expressed by the other.

### Resilience and Adaptation to Change in the Arctic, Alaska, Japan, Norway

Arctic and subarctic fisheries are a major activity in the United States, Norway, and Japan. Arctic and subarctic seas are experiencing rapid climate and other environmental change, which will affect and could potentially disrupt many of the region’s fisheries. This project brought together physical, biological, and social scientists (including author Huntington) from the three countries to examine what is known about the fisheries and ecosystems in the region and what can be said about the likely trajectories of change and their effects. In addition to discussions among the scientists, a stakeholder meeting was held in each country, involving individuals from various parts of the fisheries sector, including fishers, fishery managers, fish marketers, grocers, transporters, marine service providers, and others. One goal of these meetings was to identify the kind of information that would be useful for these stakeholders as they make decisions. Although some of the scientists and stakeholders knew one another, both groups were large (20+) and most individuals had not met before, so we consider the collaborative relationships to be in the early stage. Context knowledge, however, was high. Most of the scientists had worked with stakeholders and many of the stakeholders had worked with scientists on research projects or through fisheries management systems. With only one meeting in each locale, engagement intensity was low. Based on interactions at the meetings, scientists gained a better recognition of the time scale of useful information for the various stakeholders. For example, fishers are not looking to scientists to provide information on where to fish this year, nor are they particularly interested in mid-century predictions of sea ice cover, but are instead aided by information about likely trends in fisheries in the next decade or so, during which period they may make major investments in fishing vessels or gear or choose to buy or sell fish catch shares. The project’s engaged participation strategy was primarily aimed at—and achieved—a series of *conceptual* impacts: scientists gained new insights about the temporal scales relevant to fisheries practitioners, and fisheries practitioners increased their understanding of how climate change may impact the future of their livelihoods. We recognize that this project is different the others we present here since it was built around convenings rather than specific scientific goals. However, we include it because it met our minimum threshold for engagement, generated real-world outcomes, and allows us to consider a project with a large geographic scale that intended to influence an important scientific agenda.

## Discussion

### Relationship Maturity, Engagement Intensity, and Context Knowledge in Practice

The project case summaries above illustrate how the factors we have chosen to examine played out in practice. Both the Clyde River and Hopi projects suggest that a team can increase engagement intensity as a way to build context knowledge. In the case of the Hopi project, the engagement strategy also helped build collaborative relationships that have persisted well beyond the life of the discrete project described here. It is notable that within the first two years of the Hopi project the team did not achieve instrumental outcomes (a clear goal of the Hopi DNR partners), but by the fourth year information developed by the collaborative team was used by the tribal government to make a decision about removing livestock from drought-impacted ranges (Ferguson et al. 2016b). This result is in line with Kothari et al.’s observation that more mature collaborative relationships are more likely to result in instrumental outcomes. It is also notable that the Clyde River project achieved instrumental outcomes early in the collaboration, a result we believe can be at least partially attributed to the pre-existing relationships within the collaboration, which among other things helped the academics argue successfully for the funding needed to provide data from the weather stations to Clyde River residents in near-real time. The Northwest Arctic Borough project suggests that even without pre-existing individual relationships a collaborative group that comes together with relatively high context knowledge can utilize a high-intensity engagement strategy to achieve outcomes in a short period of time. By building common cause around the project’s goal, the group was able to come to consensus on an agenda that reflected what both the scientists and the communities valued, which in turn led to research being done that reflected those priorities. These three cases suggest avenues for further study about how high-intensity engagement may be useful to make up deficits in context knowledge and/or a lack of existing collaborative relationships. These cases also point to future research that examines the extent to which instrumental outcomes are overvalued early in a collaboration when more conceptual outcomes are more realistic and potentially more valuable for long-term impact. Our cases do suggest, though, that high-intensity engagement may be a key factor to include in projects for whom instrumental outcomes are an explicit goal.

The two cases that employed a relatively low-intensity engagement strategy, on the other hand, provide a window into how the specific and collaboratively agreed-upon goals of a project can be a meaningful benchmark against which to measure impact of an engaged participation project. In both of these cases, it is important to emphasize that each still utilized what we consider engaged participation approaches with shared governance, equitable input from both the research and practitioners involved, and outputs relevant for all involved. These are not examples of science talking to society in the traditional mode, but rather examples of science engaged in dialog with social actors in an effort to genuinely collaborate on a shared agenda.

In the case of the Colorado River study, the question at the heart of the project emerged from a shared interest in the relationships between air temperature and streamflow among the researchers and the practitioners. That shared interest was in part a product of long-lasting collaborative relationships between project researchers and members of the water management community as well as relatively deep context knowledge across the project team. The goal of the project was never to develop results that could be instrumentally used by water resources practitioners. Rather, the project team set out to answer questions that were relevant to that practitioner community in order to advance collective knowledge about system behavior, a conceptual outcome.

In a similar way, the Arctic fisheries project was not designed as an exercise in developing directly usable datasets or decision support tools for the practitioner communities that were engaged. Rather, the project was built around the recognition that the research and practitioner communities needed to learn from one another directly both for the scientists to ask more relevant questions and for the practitioners to have more realistic expectations of the science. In this project the high context knowledge of those involved may have been an important factor in mitigating any shortcomings that could have arisen from the absence of prior collaborative relationships among the individuals involved.

These two examples of projects deploying relatively low-intensity engagement are insufficient to make any empirical claims about causality, though they do suggest questions for future inquiry. For example: To what extent do long-term relationships and shared context knowledge allow for a relatively low engagement strategy to succeed in achieving project goals and outcomes? In what situations does high context knowledge by project participants provide a sufficient foundation for an engaged participation project to achieve positive early outcomes?

### Considerations for Designing Engaged Participation Projects

**Fig. 1:**
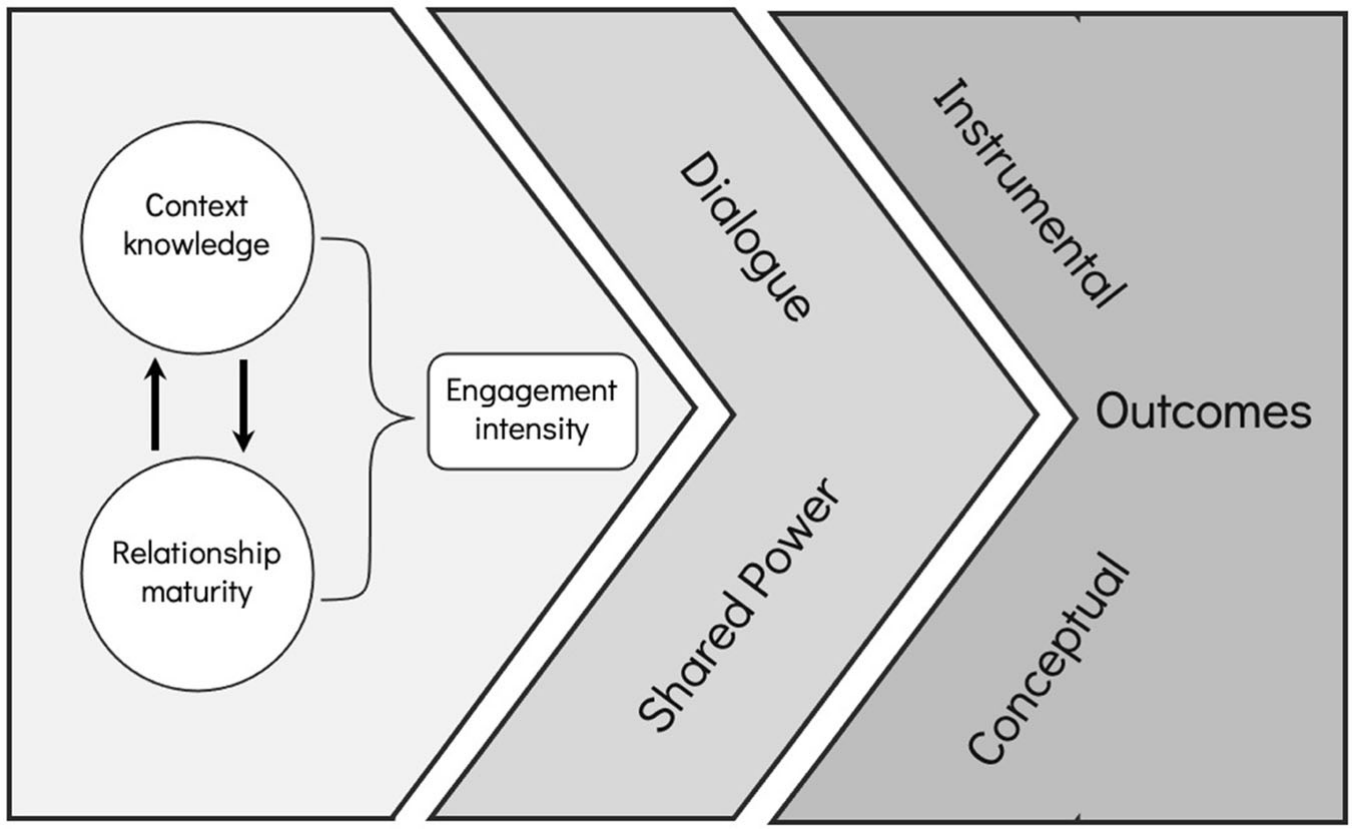
Considerations for engaged participation. Researchers can assess context knowledge and relationship maturity and adjust engagement intensity accordingly. As the project is carried out, a commitment to dialog and shared power is also necessary to achieve robust conceptual and instrumental outcomes

Our review of the literature and our cases led us to develop a process for considering how to conceptualize an engaged participation project at the outset (Fig. 1). The first step is to assess both the context knowledge of the proposed team—researchers and partners alike—and the maturity of the relationships within it. We believe those two factors are critical in deciding on the intensity of engagement that is required to first establish and then meet the shared goals of *all* participants. Once those capacities are considered and accounted for, any engaged participation project must commit to ongoing dialog and shared power (as we discuss above) so that the outputs and outcomes that emerge from the work are equitable—that is, all participants benefit—and therefore have the greatest chance for social as well as scientific impact.

### Based on This Process, We Offer the Following Recommendations

#### Consider the interactions among relationship maturity, context knowledge, and engagement intensity

These factors are not independent; they can and do interact with each other. For example, higher context knowledge increases the likelihood that participants have met or have colleagues in common, which might make their relationship look more like a “mature” partnership even if they have not worked together directly before. This may have been a factor in the Arctic fisheries project. That network effect may be a pathway to building trust if a researcher or practitioner has been recommended by colleagues. Similarly, a project that designs a high-intensity engagement strategy seems likely to speed the maturing of relationships because of the sheer amount of interaction, a phenomenon that was likely at play in the Hopi project. The factors are not substitutable, in that (for example) a mature relationship does not lead directly to the same outcomes as high engagement intensity. They can, however, complement one another, as explained next.

#### Honestly and critically assess the status of these three factors at the project design stage

We do not offer these factors as ingredients in a recipe for successful engaged participation, but rather as aspects of a collaborative undertaking that are relatively easy to assess and that can prompt conversations among project participants as they decide what will work best in their particular case. For example:

- Researchers with relatively low levels of context knowledge and limited practitioner relationships may choose to increase engagement intensity as a way to maximize the likelihood of impacts.
- Researchers with few resources for high-intensity engagement (for a particular project) could choose to focus on questions and communities about which they have or can engage others who have relatively high context knowledge and existing relationships.
- Researchers with low context knowledge can choose to draw on knowledge from other sources by bringing on new team members or collaborating with a relevant boundary organization (Guston 2001; Hellström and Jacob 2003).

#### Articulate clear and realistic goals for the engaged participation aspect of a project

If a project is designed to have significant instrumental impacts, researchers should consider whether they possess relatively mature starting relationships, high context knowledge, and resources for a relatively high-intensity engagement strategy. If those high levels are not present, the outcomes of the project should be reassessed or the team should work to increase them. Of the three factors, the intensity of engagement is the one that is almost completely within the control of the collaborative team. Our cases suggest that designing a project to utilize high-intensity engagement can go a long way toward increasing the odds of mutually acceptable outcomes even when context knowledge is relatively low and/or pre-existing collaborative relationships are few or absent. Conversely, our cases also suggest that if a project is built on mature relationships and high context knowledge, societally relevant outcomes are achievable even with relatively lower engagement intensity.

#### Consider how to engage all career stages

We recognize that senior researchers may have more mature relationships due to the time they have invested as well as more context knowledge through experience and greater access to the resources needed for high-intensity engagement than is the case for most junior researchers. However, this work requires that researchers in all stages of their career, even as graduate students and junior faculty, pursue engaged participation to the extent possible. Mentors are a particularly important source of help – but cannot remain the sole source. An important aspect of the institutional change needed to help TDA become broadly adopted by researchers is a more concerted effort to train students, provide tangible support to more junior colleagues, and generally ensure that experiential knowledge and connections are shared with up-and-coming generations of colleagues (Rozance et al. 2020). Bammer et al. (2020) point out that integrative research requires expertise in research integration and implementation as well as topical expertise, what they refer to as “knowing how” vs “knowing that.” Although we did not explicitly assess our cases to understand the extent to which “knowing how” experts were present, each does have researchers (and in some cases practitioners) who have experience with engaged participation. It is incumbent on these more experienced researchers and practitioners to support their less experienced colleagues – formally and informally - so that many more people have the opportunity to develop research integration and implementation expertise.

### Limits of Our Argument

Our assessment allowed us to identify and deeply consider the three factors we have described here, although we acknowledge two limitations to what we present. First, these five projects are not representative of all environmental research projects that choose to pursue engaged participation strategies, though our review of the literature offers some confidence that the three factors we identify are relevant to a wide range of undertakings. While we cannot draw broad generalizations about all engaged participation work from these projects alone, they are diverse enough to allow us to illustrate our key factors in a variety of contexts. Second, we did not rigorously test the role that the three factors played in each case, nor the relationships between the factors and the kinds of outcomes achieved. As we noted above, our goal was to find logical connections between projects that displayed societal impacts and factors that cross-cut those projects. What we offer are reasonable explanations to test in future research. In the meantime, what we present here can be useful for researchers who are interested in developing engaged participation projects to consider as they plan projects.

## Conclusion

During her tenure as president of the American Association of the Advancement of Science, Jane Lubchenco argued that a new social contract for science was needed to match the seemingly overwhelming environmental challenges facing the world (Lubchenco 1998). She argued that scientists should commit to devoting their energies and talents to the most pressing environmental problems of the day. Meeting this challenge will require—in addition to the energies and talents of individual scientists—changes in our institutions that will allow scientists to more fully and productively engage with society. We believe that engaging with and supporting younger researchers to take on work that uses TDA, encouraging more researchers to genuinely take on societally relevant questions, and designing and undertaking work that generates demonstrable outcomes can help accelerate the institutional changes that have proven elusive. Among the many challenges that lie in the path toward those institutional changes is the lack of focused understanding of how research that engages with the broader society actually succeeds and what success means, beyond narrow conceptions of scientific advancement and economic gains. It is also imperative to move beyond a simple dichotomy between disciplinary and transdisciplinary outcomes and recognize a range of productive modes of research that lie in between. We see enormous promise for progress across a range of endeavors, for example in proposals by the Association of Public and Land Grant Universities (2019) to support and encourage public impact research; new efforts on the part of the National Academies of Science, Engineering, and Medicine (2020) to re-envision promotion and tenure processes; and through international examples, such as universities in the Netherlands, of academia broadening its evaluation metrics to include a range of societally important skills and expertise (VSNU et al. 2019). While our case studies are connected to the environmental sciences, the lessons we draw here may be applicable to transdisciplinary efforts in many other fields as well. By empowering a larger and more diverse community of researchers to embark on the engaged participation pathway, we hope to welcome a larger and more diverse community of researchers and students in the work of engaging with society to find the currently elusive solutions to pressing environmental problems.

## Supplementary information

Supplementary Information

## Footnotes

1 For recent reviews see, for example, Norström et al. (2020), Knapp et al. (2019), Meadow et al. (2015), Harris and Lyon (2014).

2 Please see S1, included as supplemental information, for the qualitative case template.

3 Our refined definition is below, but at the assessment stage of the project we briefly characterized the extent to which the partners had experience with and knew about the contexts in which their partners work.

4 For those who are interested in the qualitative data used to develop these factors, data requests can be sent to the corresponding author, Dan Ferguson.

5 Though distinct in the way we utilize it here, our concept of context knowledge is similar to the American Evaluation Association’s Guiding Principle of competence, which includes ensuring that an evaluation team collectively possesses or seeks out the competencies necessary to work in the cultural context of their evaluation project (American Evaluation Association 2018).
