# Supplementary material for: Making a Difference: Planning for Engaged Participation in Environmental Research
Source: Environ Manage. 2022 Jan 9;69(2):227–43. doi: 10.1007/s00267-021-01585-5 (PMC8789721; doi:10.1007/s00267-021-01585-5)
Supplement: Supplementary file 1 — Supplementary Information [file 267_2021_1585_MOESM1_ESM.docx]

**Making a Difference: Planning for Engaged Participation in Environmental Research**

Daniel B. Ferguson, Alison M. Meadow, Henry P. Huntington

S1: Case Study Template

| Background | Who was involved? |
| --- | --- |
|  | Who initiated? |
|  | What was the primary question and/or issue? |
|  | Project start |
|  | Project end |
| Maturity of Relationships | Did key project participants know each other prior to the start of this project? |
|  | Have key project participants worked together prior to the start of this project? |
| Intensity of effort | Consultation details |
|  | Nature of communications |
|  | Frequency of communications |
|  | Jointly convened meetings? |
|  | Joint meetings detail |
|  | Advisory committee? |
|  | Advisory committee details |
|  | Did researchers attend stakeholder meetings? |
|  | Researchers at SH meeting details |
|  | Did stakeholders attend researcher meetings? |
|  | Stakeholder at researcher meetings details |
|  | Direct input to policy? |
|  | Policy detail |
|  | Did researchers have formal engagement training? |
| Prior context knowledge | Did researchers have prior engaged experience? |
|  | Researcher prior experience details |
|  | Did stakeholders have prior engaged experience? |
|  | Stakeholder prior exp detail |
|  | Were the researchers part of a boundary org? |
|  | Researcher boundary organization detail |
|  | Were stakeholder part of a boundary organization? |
|  | Stakeholder boundary organization detail |
|  | Were there researchers with relevant prior career practitioner experience? |
|  | Research prior career details |
|  | Were there stakeholder with relevant prior career academic experience? |
|  | Stakeholder prior career details |
|  | Approximately how long were the researchers working in region? |
|  | Approximately how long did it take to develop a common language for the project? |
| Outputs/outcomes | Research outputs |
|  | Stakeholder outputs |
|  | Outcomes for researchers |
|  | Researcher perception of stakeholder outcomes |
